# The impact of implementing a fall prevention educational session for community‐dwelling physical therapy patients

**DOI:** 10.1002/nop2.165

**Published:** 2018-06-19

**Authors:** Lynda Dee Ott

**Affiliations:** ^1^ Valdosta State University College of Nursing & Health Sciences Valdosta Georgia USA

**Keywords:** community‐dwelling adults, fall prevention, fall education, Health Belief Model, patient education, physical therapy

## Abstract

**Aim:**

The aim of this study was to evaluate the impact of a fall prevention educational session on fall risk knowledge, use of fall prevention interventions and the number of falls in community‐dwelling older persons attending physical therapy.

**Design:**

This pilot study used a mixed method design consisting of a quantitative pretest–posttest quasi‐experimental design followed by a qualitative interview.

**Method:**

An educational intervention was given with pre‐ and posttest questionnaires to determine the outcome measures of: (a) fall risk knowledge; (b) number of participants implementing fall prevention techniques; and (c) the number of falls sustained for 60 days post the educational sessions. The Health Belief Model served as the theoretical underpinnings for development and presentation of two educational sessions.

**Results:**

Eight of 20 participants completed the fall prevention educational sessions and subsequent evaluation. An increase in fall risk knowledge (*p *=* *0.031) and implementation of fall prevention techniques was noted. One fall was sustained 60 days after therapy discharge.

**What does this research add to existing knowledge in gerontology?**

- This pilot study provides a foundation for future studies to assess the importance of education in increasing fall risk knowledge and its subsequent effect on implementing fall‐preventing strategies and fall reduction in the community‐dwelling, older adult population.
- Numerous studies address fall prevention. This study facilitates collaboration between healthcare providers and physical therapists in the community setting.

**What are the implications of this new knowledge for nursing care with older people?**

- Engaging clients in conversations about fall prevention strategies that are unique to the patient can improve fall risk awareness.
- Allowing the clients to be active participants in the fall prevention strategies, addressing perceived barriers and providing supportive community resources can change the clients’ health beliefs. This change promotes the implementation of fall prevention strategies and reduces falls.

**How could the findings be used to influence policy or practice or research or education?**

- This study can serve as a guide to initiate conversations between healthcare providers and clients regarding falls. By assessing the awareness of fall risk knowledge, the provider is then able to cater education towards the patient's needs.
- The older adult population sees several types of healthcare professionals on a regular basis. By implementing collaborative fall prevention programmes, the patient is exposed repeatedly to various strategies to prevent falls by multiple persons who provide both support and accountability.

## 1 INTRODUCTION

Globally, the older adult population is expanding exponentially as the number and longevity of this population increase (National Institute on Aging, [NIA] National Institute of Health [NIH], & World Health Organization [WHO], 2011). Additionally, an estimated 28%–38% of this older adult population has had at least one documented fall (World Health Organization, & Ageing and Life Course Unit, 2007). In the United States (US) by 2030, an estimated 49 million falls will cause over 12 million injuries (Dellinger, 2017). As the growth trend continues, falls are and will continue to cause a significant burden on societies. For this study, falls are defined as an accidental and unforeseen occurrence resulting in a sudden, unexpected change in altitude of an individual landing on the ground or a lower level without loss of consciousness (Thurman, 2008).

Falls induce a myriad of physical symptoms such as an increase in injury (Garcia, Marciniak, McCune, Smith, & Ramsey, 2012; Roe et al., 2009); impaired mobility (Dykes, Carroll, Hurley, Benoit, & Middleton, 2009; Roe et al., 2009); restricted physical activity and function (Dykes et al., 2009; Moore et al., 2010); hospitalization and/or nursing home placement (Hosseini & Hosseini, 2008; Mackintosh, Hill, Dodd, Goldie, & Culham, 2005; Moore et al., 2010; Roe et al., 2009); and morbidity and mortality (Hosseini & Hosseini, 2008; Moore et al., 2010; Roe et al., 2009). In 2014, approximately 2.8 million older Americans were evaluated by emergency rooms for fall‐related injuries. Of those evaluated, nearly 27,000 older adults died due to injuries sustained from falls (Bergen, Stevens, & Burns, 2016). Stevens and Rudd (2014) identified head injuries, hip fractures and circulatory disorders as primary contributing factors in fall‐related deaths.

Economically, falls impose a significant burden on society as well. By 2040, economic global expenditures for fall‐related injuries are projected to be approximately $240 billion (World Health Organization, & Ageing and Life Course Unit, 2007). Annotated healthcare expenditure related to direct costs (medical care expense) secondary to falls is $31 billion annually (Centers for Disease Control and Prevention, 2017). Depreciated quality of life costs estimated at $162 billion encompass diminished quality of life, days absent from work, incapability to accomplish household work, restriction in earning power, consumer product injuries and adjusted years for quality of life per the US Consumer Product Safety Commission's Injury Lost Model (Zaloshnaja, Miller, Lawrence, & Romano, 2005).

According to the CDC (2017), many falls are preventable. Most falls are related to multiple intrinsic and extrinsic factors related to behavioural, biological, socioeconomical and environmental risk factors (Garcia et al., 2012; World Health Organization, & Ageing and Life Course Unit, 2007). Successful community‐based fall prevention programmes use multifactorial approaches (Panel on Prevention of Falls in Older Persons [PPFOP], American Geriatrics Society [AGS], British Geriatrics Society [BGS], 2011) aimed at addressing identified risk factors such as environmental/home hazards, medication usage, balance and gait abnormalities, vision impairment and education personalized to a person's identifiable risk (Garcia et al., 2012; Howard, Beitman, Walker, & Moore, 2016).

Fall prevention education provides numerous advantages such as improving fall prevention awareness, perception of fall prevention intervention, self‐efficacy and in some cases, a reduction in the number of falls (Brouwer, Walker, Rydahl, & Culham, 2003; Clemson et al., 2004; Deery, Day, & Fildes, 2000; Haines et al., 2011; Schepens, Panzer, & Goldberg, 2011). Fall prevention education in conjunction with fall prevention strategies can reduce falls. During an educational‐based study, Kempton, van Beurden, Sladden, Garner, and Beard (2000) annotated that participants had fewer self‐reported falls and hospitalization. These findings were attributed to an increase in knowledge regarding falls, the importance of activity, wearing safe shoes, improving balance and reducing medications that increase fall risks. Furthermore, addressing fall intervention facilitators and barriers enhance compliance (Dickinson et al., 2011). Fall prevention education should encompass information regarding falls and available services, annotate easy accessibility to interventions, consider recipient's level of education, cultural and activity levels and highlight the beneficial outcomes of preventing falls (Dickinson et al., 2011). Fall prevention education is an economical and beneficial tool for reducing fall incidences.

Short‐term fall prevention programmes are effective in preventing falls (Howard et al., 2016). Furthermore, Verghese (2016) identified that fall risk awareness is related to the use of fall prevention strategies. Limited data are available on collaborative nurse practitioner and physical therapist led fall prevention educational sessions in relation to fall risk awareness in community‐dwelling individuals. The primary goal is to evaluate the impact of the educational sessions composed of an initial nurse practitioner led, personalized fall prevention educational session followed by a second physical therapist led session with clients identified at risk for falls attending physical therapy on increased awareness of fall risk in community‐dwelling older adults. Additional objectives are to determine if the sessions prompted individuals to implement any fall prevention interventions suggested by the nurse practitioner and physical therapist and thereby reduce falls.

The Health Belief Model (HBM) is the theoretical foundation for this study. First developed by US Public Health Service social psychologists, Hochbaum, Rosenstock and Kegels in the 1950, the HBM is commonly used for health promotion because of the identified correlation between personal beliefs and implementation of health behaviours (Current Nursing, 2012; Hayden, 2009). The HBM facilitates an understanding of the individual's health behaviour, possible reasons for noncompliance and interventions to facilitate a behavioural change (Rosenstock, Strecher, & Becker, 1988). The HBM constructs evaluate an individual's perception of the seriousness and/or severity of falls, fall risk factors, benefits of implementing fall interventions and identified barriers. The educational sessions were developed to address individual perceptions, modifiable factors and likelihood of implementing a fall prevention intervention (Hayden, 2009).

## 2 METHODS

### 2.1 Setting and participants

A convenience sample from community‐dwelling individuals who were referred to or attending physical therapy (PT) at a privately owned clinic in Southern Georgia was recruited to participate in this pilot study. Persons who (a) aged 18 and older; (b) lived in a private residence or independent‐living senior facility; (c) met the American Academy of Neurology's (AAN) guidelines for assessing fall risks in patients (Thurman, 2008); (d) was able to ambulate with or without assistive devices; and (e) was able to attend outpatient physical therapy sessions and educational intervention were included in the study. Participants were excluded if they scored less than 18 on the Montreal Cognitive Assessment's (MoCA) basic examination (written permission granted by Tina Brosseau in personal correspondence), non‐English speaking individuals without a translator present, or if they did not meet the inclusion criteria.

### 2.2 Measures

The Fall Risk Awareness Questionnaire (FRAQ) initially developed in 2002, determined the awareness of fall risk factors in clients. FRAQ Version 2.0 was released in November 2011. The FRAQ was composed of four sections of inquiry: (a) assesses patient's cognizance of fall‐related issues in an open‐ended format, (b) quantifies patient's fall risk factors knowledge, (c) identifies patient's demographic information and fall risk and falls history and (d) allows for patient input on questionnaire improvements (C. Sadowski, FRAQ guide personal communication, January 30, 2012). The primary focus of the questionnaire centred on the quantifiable risk factor knowledge identified by the clients. Wiens, Koleba, Jones, and Feeny (2006) annotated the questionnaire had been validated in a variety of patients and settings such as acute care, outpatient, community and long‐term care settings. The FRAQ demonstrated construct validity and with multiple implementations, reliability (Wiens et al., 2006). This tool allowed the investigator to identify the changes in the previous identified sections of the FRAQ by comparing pretest and posttest data and to collect demographical information.

Furthermore, the two questionnaires developed by Hill et al. (2009) were administered to identify the clients’ perceptions of the likelihood of falls, severity of fall injuries, knowledge of fall risk factors and prevention techniques, motivation for fall prevention implementation and the assessment of fall prevention interventions usage at home (Permission given by personal communication to adapt questions as necessary for the study, February 6, 2012). Additionally, Lord, Sherrington, and Menz's (2001) questionnaire identified the number and etiology of falls the patient had sustained during the 30 and 60 days following physical therapy discharge (permission given to use via personal communication January 11, 2012).

The final tool was a flow sheet to maintain continuity and successful completion of tasks. The Falls Prevention Checklist (see Supporting information section) development by the principal investigator facilitated monitoring of tasks for the project while outlining educational curriculum. This checklist provided a simple, efficient way to monitor the programme's completion progress throughout the course of the study to ensure consistency of implementation.

### 2.3 Design

This study used a mixed method design consisting of a quantitative pretest–posttest quasi‐experimental design followed by a qualitative interview. The investigator, a certified family nurse practitioner with 7 years of experience in neurology, met with the experienced, physical therapist, DPT, prior to the initiation of the study to enhance the reliability of the study. The content reviewed included the screening guidelines, educational materials and questionnaire implementation for competency.

Community‐dwelling individuals who presented to the physical therapy clinic for their initial or regularly scheduled visit were evaluated by the physical therapist. If the patient met the inclusion criteria which included inquiring about falls within the past year, reviewing history for fall risk factors and evaluating balance, gait, strength and coordination per AAN's fall risk assessment guideline (Thurman, 2008), he or she was asked to participate in the study. The principal investigator presented a brief five‐minute overview of the purpose and intent of the study. If any cognitive impairment was suspected by the principal investigator, MoCA was used. The MoCA, developed by Dr. Ziad Nasreddinethe, was validated in its ability to distinguish mild cognitive impairment from normative processes (Nasreddine et al., 2005). After signing the consent, administration of the Falls Risk Awareness Questionnaire [FRAQ] (Wiens et al., 2006) provided a baseline (pretest) evaluation of knowledge regarding fall risk factors. Educational needs were determined by the participant's independent level of functioning, comorbidities and FRAQ scores. The initial educational intervention was presented at convenient times for the patient, family members and/or caretakers. The educational information developed by the investigator consisted of a PowerPoint presentation summarizing the definition of a fall (Thurman, 2008), prevalence of falls (Dellinger, 2017; World Health Organization, & Ageing and Life Course Unit, 2007), complication of falls (Dykes et al., 2009; Garcia et al., 2012; Hosseini & Hosseini, 2008; Mackintosh et al., 2005; Moore et al., 2010; Roe et al., 2009; World Health Organization, & Ageing and Life Course Unit, 2007; Zaloshnaja et al., 2005), where older adults tend to fall the most, high‐fall risk‐associated diseases, (Learn Not to Fall, 2012) common risk factors with emphasis on pertinent fall risk factors identified for each participants and fall prevention interventions (DynaMed Plus, 2018; PPFOP/AGS/BGS, 2011). A resource booklet provided to the participant acted as a visual reminder of pertinent fall risk factors. The booklet also provided information on available community resources in addition to a home safety checklist (CDC, 2005; Veterans Affairs National Center for Patient Safety, 2012), information on how to prevent falls (CDC, nd), common myths associated with falls (National Council on Aging, 2012) and *The Falling LinKS Toolkit* (Radebaugh et al., 2011). The first educational session lasted approximately 90 min.

**Figure 1:**
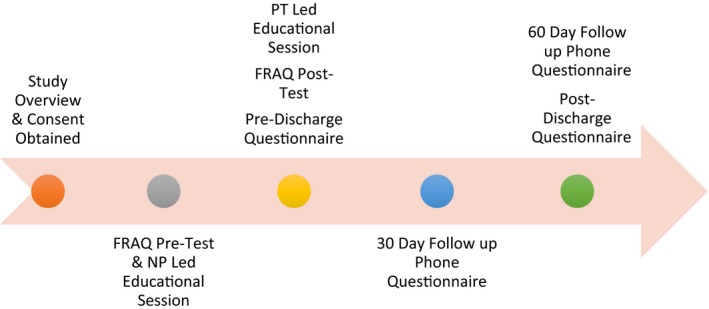
Study implement timeline

Prior to therapy discharge, the physical therapist presented the second educational session followed by administration of the FRAQ posttest. The second educational session highlighted identified fall risks for the participant and appropriate fall prevention interventions to be implemented. A modified follow‐up phone questionnaire by Lord et al. (2001) was verbally given at 30 and 60 days after the second educational session. Also, the 60‐day follow‐up phone interviews included Hill et al.'s (2009) postdischarge questionnaire. See Figure 1.

### 2.4 Data collection

After giving an overview of the study and obtaining the consent, the clients completed the 15‐min FRAQ questionnaire to establish a baseline of fall prevention knowledge followed by the first educational session. Prior to the client's discharge from physical therapy, a second educational session was conducted. Next, the FRAQ questionnaire was readministered to identify if any change in the knowledge base had occurred. In addition, Hill et al.'s (2009) predischarge questionnaire was used with a completion time range from 10 to 15 min. Lord et al.'s (2001) 5‐ to 10‐min questionnaire was used to perform the 30‐day follow‐up phone interview. The final data collection was obtained by using Hill et al.'s (2009) post discharge questionnaire and Lord et al.’ (2001) follow‐up questionnaire via a phone interview. The entire phone interview was approximately 15–25 min in duration.

### 2.5 Data analysis

Data analysis was completed by using SPSS Version 21.0 (IBM, Chicago, IL, USA) (IBM Corp., 2012). A two‐tailed paired *t* test was used to compare the means scores of the FRAQ posttest intervention total scores to the FRAQ pretest intervention total scores. For qualitative analysis, the survey responses were recorded verbatim by the primary investigator then coded using qualitative description. The responses were summarized into themes and presented as quantifiable data by the number and percentages identified (Hill et al., 2011). A review of data and themes were completed by the physical therapist and a second reviewer to evaluate for consistency and accuracy.

### 2.6 Ethical considerations

This study was reviewed and deemed exempt by the University of South Alabama's (USA) Institutional Review Board. Similarly, the project was approved by the privately owned physical therapy clinic. All participants reviewed and signed an informed consent form. The author denies any conflicts of interest.

## 3 RESULTS

Twenty clients were initially evaluated to be the participants in the study. Only 10 clients met the identified inclusion criteria. Two clients withdrew from the programme due to not completing the second educational session and transportation difficulties. The remaining eight participants met all criteria and completed the study.

### 3.1 Demographics

The patient population consisted of six females (75%). The mean age for all participants was 65 years. Seventy‐five percent (*N* = 6) of the individuals lived in a private residence with another person. Educationally, 50% (4) of participants graduated from high school with approximately 38% (*N* = 3) obtaining a vocational or technical degree. The overall health status perception was good (62.5%) with the most frequent comorbidities consisting of arthritis/rheumatism and hypertension (62.5%). Within the past 2 years, three participants had fallen without injury while one participant sustained only “mild injuries.” Two of those falls were within the last year (Table 1).

**Table 1:** Patient population characteristics

Characteristic	*n* = 8	Mean
		*n* (%)
Maturity in years	Age	65
Sex	Female	6 (75%)
Living arrangements	With another person	6 (75%)
	Alone	1 (12.5%)
	Lives with two or more persons	1 (12.5%)
Residence	House	6 (75%)
	Mobile home	1 (12.5%)
Perception of health status	Good	5 (62.5%)
	Fair	3 (37.5%)
Prior falls	In the last 6 months	1 (12.5%)
	In the last year	1 (12.5%)
	Greater than 1 year	2 (25%)
Level of education	High School Grad	4 (50%)
	Non‐University Degree (Vocational/Technical)	3 (37.5%)
Utilization of assistive device	None	5 (62.5%)
	Walker	3 (37.5%)
Comorbidities	Arthritis or rheumatism	5 (62.5%)
	Back problems	4 (50%)
	Hypertension	5 (62.5%)
	Diabetes	3 (37.5%)
	Effects of stroke	3 (37.5%)
	Difficulty controlling bladder	3 (37.5%)
Medications	Diuretics	3 (37.5%)
	Blood pressure	5 (62.5%)
	Anti‐inflammatories (NSAIDs)	5 (62.5%)
Acknowledges they have fall risks	Yes	8 (100%)

### 3.2 Fall risk awareness

**Figure 2:**
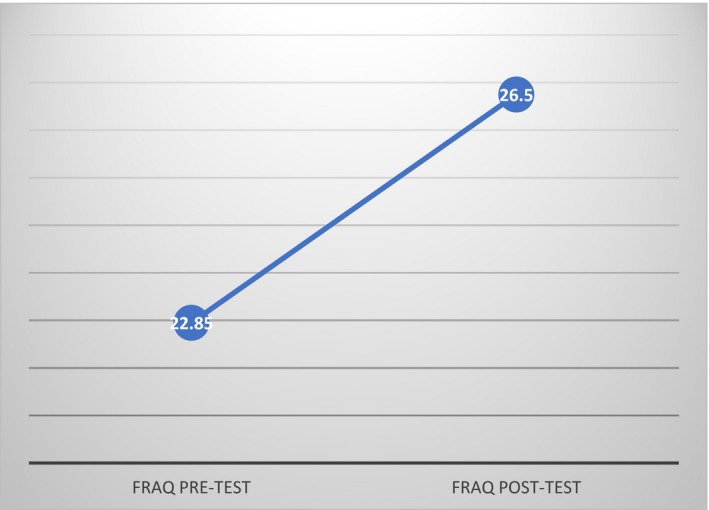
FRAQ Scores

A statically significant difference was noted in the means of the pretest and posttest interventional FRAQ scores (*p* = 0.031). The mean value of the participants’ pretest–posttest score was 0.096 (22.85–26.5). See Figure 2. A review of the individual FRAQ themes identified an increased awareness of medications related to fall risks by having a 50% increase in scores from pretest to posttest.

### 3.3 Implementation of fall intervention

Overview of the 30‐ and 60‐day questionnaires identified two participants felt they would fall within the next 6 months and three were undecided despite having completed the intervention. Five participants felt that they would most likely fall in the early morning hours while going to the bathroom or kitchen. Multiple home intervention strategies to reduce falls were identified by the participants. Removing throw rugs and clutter were the primary interventions verbalized by the participants where they felt that they could implement at home to reduce fall risks. Seventy‐five percent (*N* = 6) identified that they could reduce their risk of falling if they implemented one or both home modifications. Sixty‐two percent (*N* = 5) of the participants verbalized that they were motivated to implement a home intervention strategy. Fifty percent (*N* = 4) of the participants have made lifestyle changes based on their strategies. Two participants (*N* = 2) were planning on implementing the home intervention but had not done so due to needing assistance with the modification such as having railing put on the porch or grab bars for the shower. One participant named multiple fall prevention strategies such as clearing clutter, waiting before getting out of bed and stabilizing bathroom mats; however, when he or she was asked about why they had not tried these interventions at home the response was, “I don't feel they are applicable.”

All participants felt that they would benefit and could participant in balance and strengthening exercise but only four participants continued participating in regular exercise classes after discharge from physical therapy. Two of the nonexercising participants had medical reasons for not continuing with strengthening: one had back surgery and the other was in a motor vehicle accident and instructed not to exercise at the time. Six individuals (75%) have had their eyes examined within the last year. However, most participants did not buy new walking shoes, talk to primary care providers regarding medications that increase fall risks, or feel as if they have any difficulty with showering or getting dressed. When asked if they had any comments on prevention of falls in the home for older people, most did not have any comments or simply said to “use common sense” and “just be careful.”

### 3.4 Falls

This pilot study's overall objective was to decrease the number of falls sustained in community‐dwelling individuals. Prior to the initiation of the project, four persons had fallen for the last 2 years. Two falls were sustained for the last 12 months or less. The post 30‐day follow‐up interview did not identify any falls since the last educational session. At the 60 days post education follow‐up phone interview, one participant (12.5%) experienced a fall without injuries in his yard after his knees “gave way” on him while watering his plants.

## 4 DISCUSSION

The purpose of this study was to evaluate the impact of a fall prevention educational intervention on fall risk awareness, implementation of fall prevention strategies and falls sustained. Various frameworks and models are implemented to ensure success with measurement outcomes. Choosing the most appropriate theoretical underpinnings is essential for educationally focused projects. The HBM (Rosenstock et al., 1988) facilitates the development of an educational programme promoting fall reduction lifestyle changes. Various components of the HBM incorporate clients’ point of views when considering severity, frequency and perceived risk factors of falls. Plus, potential benefits and perceived barriers to fall prevention interventions are identified. A change in health behaviours occurs by the patient's beliefs and perceptions of seriousness, susceptibility, benefits and barriers to a behaviour (Current Nursing, 2012; Hayden, 2009; Rosenstock et al., 1988). The educational component is tailored to the individual recipients, contains attainable outcomes and promotes independence to increase adherence (Miller, 2010; Nyman & Yardley, 2009). Use of a combination of written information, a PowerPoint presentation and an oral presentation increases information retention (Miller, 2010). The FRAQ (Wiens et al., 2006), the 30‐ and 60‐day telephone questionnaire (Lord et al., 2001) and the predischarge and postdischarge questionnaires (Hill et al., 2009) all have the foundation underpinnings of the HBM by facilitating the identification of knowledge, perceived susceptibility, risks and view of fall intervention techniques.

Enhancing awareness begins with education. Assessing clients’ knowledge deficits is the initial steps to providing individualized, pertinent education. Focused and personalized approaches to education lead to increasing the desire to implement behaviour changes (Hill et al., 2011). The increase in fall risk awareness as demonstrated by improved FRAQ scores supports these findings. An increase in the number of participants who are currently implementing some type of fall prevention technique in conjunction with a decrease in falls sustained is noted. These findings are consistent with previous studies showing that an increase in awareness in conjunction with identifying and educating clients, family members and caregivers on fall risk factors specific for each patient prior to discharge facilitates better fall prevention outcomes in the community setting (Clemson et al., 2004; Institute for Healthcare Improvement, n.d.).

## 5 LIMITATIONS

Various limitations exist for this study. Future research needs to be completed with a larger sample size, a more in‐depth analysis of the relationship between an increase in fall risk awareness and the motivation to implement fall prevention strategies. The recruitment process was based on the client population being referred to the private physical therapy clinic by local healthcare providers which limits the generalizability. Two barriers were identified by the physical therapy clinic; clients either did not have adequate transportation or finances to attend therapy. Having a small, patient population limited the generalizability of the study. Using follow‐up phone interviews to collect data instead of face‐to‐face interviews is difficult at times. Problems with phone reception, participants’ busy schedules, environmental distractions and participants with hearing difficulties made accuracy challenging. Future projects may consider follow‐up questions to be given at a primary care or specialty office in person. This study addressed education barriers; however, future studies need to address those barriers that prevent participants from implementing fall prevention techniques at home. Additionally, changes in the methodology to consider are to have a longer timeframe for evaluation and follow‐up with a minimum of 3 months and to perform a randomized clinical trial. Various locations for implementation of future studies are in primary care facilities, in‐patient rehabilitation centres and senior citizen facilities. Finally, the study would benefit from having a home evaluation and resources available to help implement or install recommended changes.

## 6 CONCLUSION

The purpose of this pilot study was to appraise the effectiveness of a two‐session educational intervention on fall risk awareness and its impact, if any, on implementation of fall risk prevention techniques and a reduction in falls. This study demonstrates that an educational intervention within this older adult population attending physical therapy had an increase in fall risk knowledge as identified by the increase in the post‐FRAQ scores. Post interventional findings identify that this population also had a higher number of participants using fall prevention interventions at home with a reduction the number of falls noted compared with previous years. Most falls are foreseeable and avertible (World Health Organization, & Ageing and Life Course Unit, 2007). Educating clients about falls and prevention strategies is the initial step in overcoming the enormous impact falls have on society.

### 6.1 Relevance to practice

Falls are a significant problem for the healthcare system. As the population ages, the problem will only escalate. This pilot study serves as a foundation for future research. It also can guide initial conversations between healthcare providers and clients about falls in the community. Conversations and education can increase fall prevention awareness and in turn, reduce falls.

## CONFLICT OF INTEREST

No conflict of interest has been declared by the author.
